# [3+2]-Cycloaddition of azomethine ylides to 5-methylidene-3-aryl-2-сhalcogen-imidazolones: access to dispiro indolinone-pyrrolidine-imidazolones

**DOI:** 10.1098/rsos.211967

**Published:** 2022-03-09

**Authors:** Maxim E. Kukushkin, Alexandra A. Kondratieva, Nikita A. Karpov, Dmitry E. Shybanov, Viktor A. Tafeenko, Vitaly A. Roznyatovsky, Yuri K. Grishin, Anna A. Moiseeva, Nikolai V. Zyk, Elena K. Beloglazkina

**Affiliations:** Department of Chemistry, M.V. Lomonosov Moscow State University, Leninskie Gory, 1-3, 119991 Moscow, Russian Federation

**Keywords:** dispiro derivatives, 1,3-dipolar cycloaddition, azomethine ylides, 5-methylidene-2-chalcogen-imidazolones

## Abstract

A synthesis of dispiro derivatives from 5-methylidene-2-chalcogenimidazolones and azomethine ylides generated from isatins and N-substituted *α*-amino acids has been developed.

## 1. Introduction

2-Сhalcogen-imidazolones (hydantoins and thiohydantoins) are well-known classes of heterocyclic compounds with proven biological activity, characterized by a variety of approaches to their synthesis and application, which are well described in the literature [1,2]. Compounds of these structural classes exhibit a wide spectrum of pharmacological activity against various significant diseases, such as cancers, microbial infections, metabolic diseases and epilepsy [3]. Several hydantoin and thiohydantoin derivatives, such as phenytoin, nitrofurantoin and enzalutamide are currently in clinical use.

**Figure 1.**
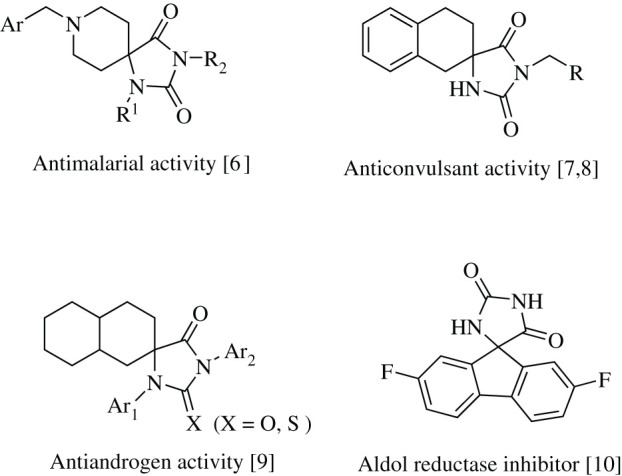
Examples of biologically active spirohydantoins and thiohydantoins.

A convenient feature of the hydantoin and thiohydantoin scaffolds is the possibility to easily introduce various functional groups into these molecules. In particular, arylmethylidene- and indolylidene-substituted thiohydantoins and hydantoins can be functionalized by 1,3-dipolar cycloaddition reactions at the exocyclic C=C double bond to obtain derivatives of interest due to their significant cytotoxicity, which is associated with the potential of inhibiting p53-MDM2 protein–protein interactions [4,5]. Furthermore, the rigidity of the spiro-fused framework makes it possible to introduce the required substituents into different sites of the molecule and thus fine-tune the structure of the resulting compounds to facilitate interactions with the selected target [4,6–10]. Examples of biologically active spiro derivatives of hydantoins and 2-thiohydantoins are shown in figure 1.

5-Arylmethylidene- and 5-indolylidene-substituted hydantoins and thiohydantoins have previously been studied in cycloaddition reactions with azomethine ylides [4,5,11]. Both 5-arylmethylidene- and 5-indolylidenthiohydantoins do not participate in 1,3-dipolar cycloaddition of azomethine ylides, synthesized from *α*-amino acids and containing substituents at the nitrogen atom other than the methyl group [5]. By contrast, the 1,1-disubstituted double bond present in the structure of 5-methylidene-imidazolones is sterically not hindered and may be suitable for various chemical transformations, including those that are impossible for more spatially hindered analogues. To date, a singular example of 5-methylidene hydantoin derivatives in 1,3-dipolar cycloaddition reactions has been examined in literature [12], while 5-methylidethiohydantoins have not been investigated. As established in this work, 5-methylidene hydantoins and thiohydantoins successfully take part in reactions with azomethine ylides derived from sarcosine, as well as α-amino acids with larger substituents at the nitrogen atom.

## 2. Results and discussion

**Scheme 1.**
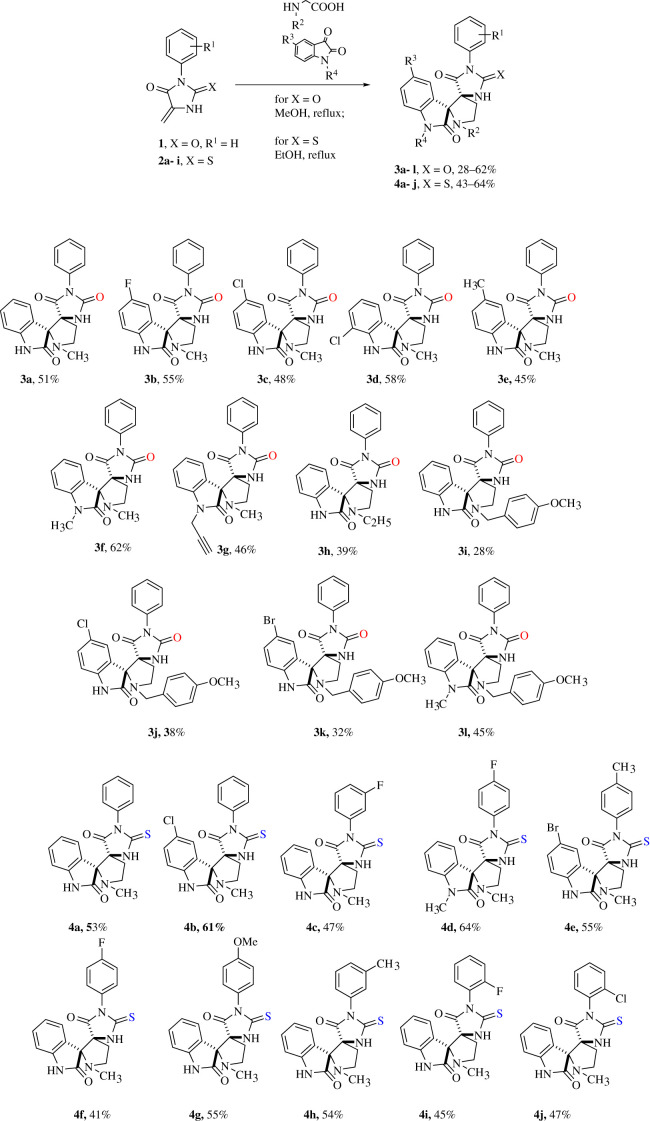
Synthesis of dispiro compounds **3** and **4** based on methylidene hydantoin **1** or methylidene thiohydantoins **2.**

In this study, we developed a regio- and diastereoselective synthesis of dispiro derivatives of 5-methylidene hydantoins and thiohydantoins based on 1,3-dipolar cycloaddition to these dipolarophiles of azomethine ylides generated *in situ* from isatins and N-substituted glycines. The general reaction scheme and structures of the obtained products are shown in scheme 1.

Initially, we optimized the reaction conditions for 1,3-dipolar cycloaddition using sarcosine and isatine as the 1,3-dipole precursors and N(3)-Ph-substituted methylidene hydantoin **1** or thiohydantoin **2a** [13] as dipolarophiles, and by varying the solvent. To avoid undesirable dimerization of methylidene derivatives **1** and **2a** [14], they were introduced into a solution preheated to the boiling temperature. The yields of the products obtained by reactions in various solvents are presented in table 1. The resulting spiro derivatives **3a** and **4a** were isolated from the reaction mixtures by column chromatography. The optimal reaction conditions were as follows: initially, sarcosine was dissolved in the respective solvent, after which the solution was heated to reflux, dipolarophile **1** or **2a** and isatin were added in one portion, and then the mixture was refluxed with stirring for 4 h.

**Table 1.** Optimization of the reaction conditions for 5-methylidene hydantoin **1** and thiohydantoin **2а** with azomethine ylides (see scheme 1; *R*_1_ = *R*_3_ = H, *R*_2_ = Me).

no.	X	solvent	yield, %
1	O	MeOH	51
2	O	EtOH	26
3	O	toluene	8
4	O	THF	trace
5	S	MeOH	38
6	S	EtOH	53
7	S	toluene	10
8	S	THF	trace

In toluene and tetrahydrofuran (THF), we did not observe complete conversion of the starting compounds **1** and **2а**, based on thin layer chromatography (TLC) data. The best yields were obtained when the reaction was carried out in methanol for spiro hydantoin **3a**, and in ethanol for thiohydantoin **4а**, thus these two solvents were used in further syntheses (scheme 1). It can be assumed that, in the case of more reactive methylidene hydantoins, the polarity of the solvent plays a more important role; therefore, the yields of the products in the more polar methanol are higher than in ethanol. Less reactive methylidentityhydantoins seem to react more efficiently with increasing temperature, and the higher-boiling ethanol appears to be a better solvent.

It was found that 1,3-dipolar cycloaddition reactions with compound **1** successfully proceeded not only with sarcosine, but also with more sterically hindered amino acids such as N-ethylglycine and N-(4-methoxy)benzylglycine (N-PMB-glycine) (scheme 1; compounds **3 h**–**l**). However, when amino acids with bulky substituents at the nitrogen atom, such as N-(4-chloro)phenylglycine, isopropylglycine and *tert*-butylglycine, were introduced into the reaction, target product **3** did not form. In contrast with N-(4-chloro)phenylglycine, N-PMB-glycine proved successful in this type of reaction, thus it can be concluded that an additional CH_2_-group is probably required between the nitrogen atom and the bulky substituent in order to reduce steric hindrance.

Azomethine ylides generated from N-substituted isatins, such as N-methylisatin can be successfully introduced into the reaction.

Comparing the yields of 1,3-dipolar cycloaddition reactions to 5-methylidene hydantoins and thiohydantoins with the previously described reactions of arylmethylidene hydantoins and thiohydantoins [4,5,11], it can be noted that the yields of reactions with methylene derivatives **1** and **2** are somewhat lower than those of the corresponding arylmethylidene analogues. This can be explained by the side reaction of dimerization of compounds **1** and **2**, which easily takes place in an acidic medium for methylidene derivatives of hydantoins [14], but is not characteristic for their arylmethylidene derivatives.

In all reactions, we observed the formation of 1,3-dipolar cycloaddition products as single diastereomers with the relative 2′R*,4S*-configuration; other possible stereoisomeric products were not detected in the nuclear magnetic resonance (NMR) spectra of the reaction mixtures, even in trace amounts.

NMR spectroscopy data confirmed the structure of the resulting products. Application of two-dimensional techniques (heteronuclear multiple bond correlation (HMBC), heteronuclear single quantum coherence (HSQC) and nuclear Overhauser effect spectroscopy (NOESY)) provided complete resonance signal assignments in the spectra of compounds **3f** and **4i** (see electronic supplementary material, information, pp. S13, S44). The observed long-range ^1^H-^13^C correlations corresponded to those of dispiro products. Thus, the HMBC spectrum of **3f** (electronic supplementary material, information, p. S13) reveals interactions of the spiro carbon, which connects the pyrrolidine and isatin fragments (*δ*C 77.5 ppm, C_6_D_6_), with methylene protons (*δ*H 3.30 and 2.50 ppm) and NCH_3_ protons (*δ*H 2.01 ppm) of the pyrrolidine ring, and *ortho*-proton of the benzoic ring (*δ*H 7.66 ppm). Additional connectivity between this carbon and the NH-group, related to the isatin was observed for compound **4i** (*δ*C 73.6 ppm, *δ*H 10.68 ppm). In addition, other spiro carbons display long-range interactions with protons of both pyrrolidine and hydantoin rings.

**Figure 2.**
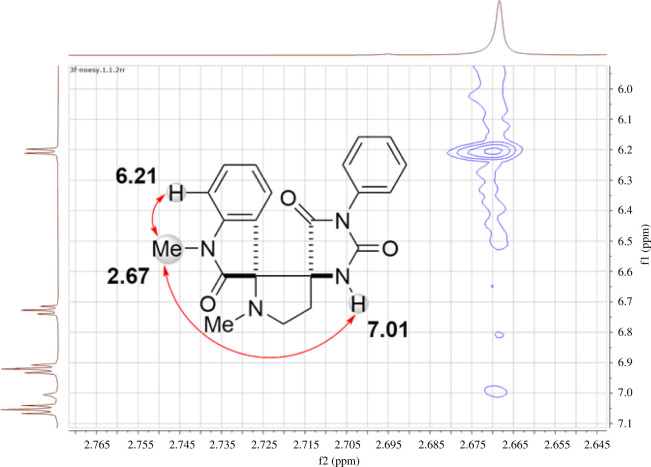
The observed correlations in the two-dimensional NOESY NMR spectrum of the dispiro compound **3f**.

The stereochemistry of the resulting products was established using the data from the NOE study for compound **3f** and X-ray structural analysis for compounds **3j** and **4b**. Thus, in the ^1^H-^1^H NOESY spectrum of compound **3f**, the cross-peaks corresponding to the interactions of the N-CH_3_ protons (*δ*H 2.67 ppm) and the proton in the *ortho*-position of the benzoic ring of the isatin fragment (*δ*H 6.21 ppm), and the proton of the NH group of the hydantoin fragment (*δ*H 7.01 ppm) were detected (figure 2). This confirms the location of these structural fragments on one side of the plane of the central pyrrolidine fragment.

**Figure 3.**
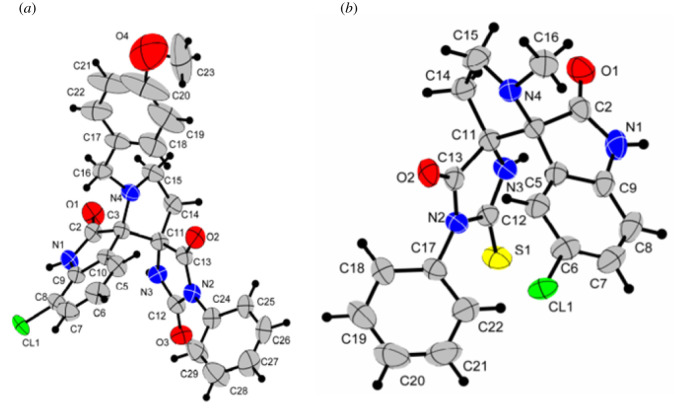
Molecular structures of dispiro compounds **3j** (*a*) and **4b** (*b*).

The structures of the obtained dispirohydantoin **3j** and thiohydantoin **4b** were also confirmed by X-ray diffraction analysis. The molecular structures of compounds **3j** and **4b** are shown in figure 3. The five-membered imidazolone rings in these molecules are practically flat; the angles between the planes of the spiro-connected imidazolone, pyrrolidine and indolinone fragments are close to 90°.

**Scheme 2.**
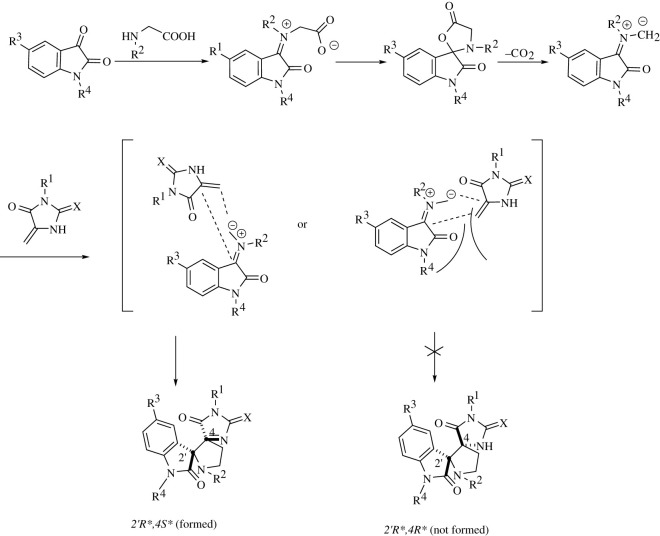
The proposed reaction mechanism.

The stereochemistry of the reaction can be explained by considering the mechanism of 1,3-dipolar cycloaddition, as shown in scheme 2. This mechanism is similar to that described for 5-arylmethylidene hydantoins and thiohydantoins [4] and includes the following stages (scheme 2): formation of the iminium intermediate as a result of sarcosine attacking the carbonyl group of isatin, the subsequent cyclization of the iminium intermediate into a spiro-lactone and the elimination of CO_2_ from the latter with the generation of the 1,3-dipole, which regio- and diastereoselectively attacks the C=C double bond of 5-methylidene-2-сhalcogen-imidazolone. It should be noted that the attack of the dipole can proceed from both above and below the double bond plane, resulting in a mixture of enantiomers. Possible routes of the azomethine ylide approach to the dipolarophile moiety are shown in scheme 2. Based on the structure of the products, we can conclude that the cycloaddition reaction takes place through the *exo* transition state [11] (scheme 2), possibly due to increase in the transition state energy, as a result of electrostatic repulsion of the cis-located carbonyl groups of azomethine ylide and dipolarophile, in the other approaches.

**Figure 4.**
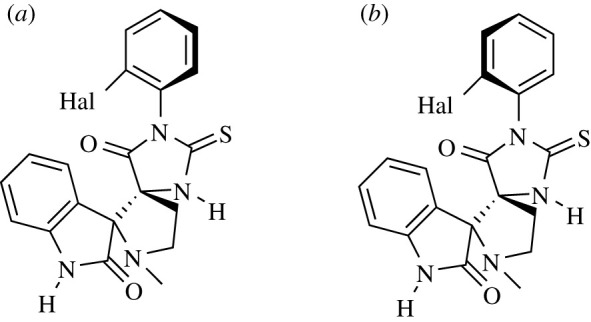
Transoid (*a*) and cisoid (*b*) configurations of axially chiral compounds **4i** and **4j**.

It should be noted that the ^1^H, ^13^C and ^19^F NMR spectra of *ortho*-substituted products **4i** and**4j** show two sets of resonance signals (electronic supplementary material, Information, pp. S43, S45 and S47).This can be explained by the hindered rotation around the single bond С(Ar)-N(imidazolone) of the hydantoin fragment, and consequently, the existence of compounds **4i** and **4j** in the form of atropisomers (axially chiral heterocyclic analogues of biaryl derivatives similar to those described for *ortho*-substituted 5-alkyl-2-thiohydantoins) [15–18]. The axially chiral compounds **4i** and **4j** may appear in stereoisomeric forms (figure 4) with a transoid (*a*) and cisoid (*b*) arrangement of the highest substituent at C5 and halogen atom in the aryl fragment of the 2-thioimidazolone cycle.

The chemical shift differences of the *ortho*-protons of the benzene ring are especially evident for the two atropisomers of compounds **4i** and **4j** (approx. 1 ppm, approx. 6.3 and 7.3 ppm), and significant differences were also observed for the protons of the NH and N-Me groups (electronic supplementary material, Information, p. S43).

The barrier of internal rotation around the N-C bond was estimated using dynamic ^1^H NMR study of compound **4i** with an atropisomer population ratio of *ca* 1 : 1.4 at ambient temperature. The temperature dependence of the proton spectra at set intervals from 22°C to 80°C corresponds to slow and intermediate rates of exchange. The line shape analysis of the N-Me-groups signals [19] (electronic supplementary material, Information, p. S45) resulted in activation barriers ΔG^≠^_300_ = 19.0 and 19.2 kcal mol^−1^ for the two directions. These values are comparable to the barrier found for *ortho*-F-substituted 2-thioimidazolone derivatives (19.8 kcal mole^−1^) [15] also using dynamic NMR study. One also draws attention to the strong temperature dependence of the chemical shifts of the NH protons of compound **4i** in DMSO (electronic supplementary material, Information, p. S45), which is most likely associated with intermolecular processes.

The question of which of the atropisomers **A** or **B** is predominant cannot be unambiguously answered based on the available data. However, some assumptions can be made; the difference in the chemical shifts of the *ortho*-protons for the two isomers of compound **4i** is about 1 ppm (electronic supplementary material, Information, p. S43), while the proton is more shielded in the dominant isomer (*δortho* 6.4 ppm for the major stereoisomer and 7.35 ppm for the minor one). It should be noted that the average value of this chemical shift (6.88 ppm, in this case we averaged without the weighting factor) is close to *δortho* = 6.84 ppm for compound **4а**, in which the rotation of the phenyl group is free. The influence of fluorine on the chemical shift can be neglected since the increment of this substituent in benzene is −0.03 ppm [20]; thus, the shielding of *ortho*-protons in the 3-aryl-2-thiohydantoin series may be considered as a common feature. It can be assumed that the influence of the magnetic anisotropy of C=S and C=O exocyclic bonds on the chemical shifts is insignificant and/or the same, since according to X-ray diffraction data (figure 3), the plane of the aromatic substituent is moved in relation to the plane of the heterocycle; however, the preferred arrangement of the *ortho*-protons in relative proximity around one of these two exocyclic multiple bonds is preserved.

It can be assumed that a significant part of the difference between the **4i** atropisomers is due to the magnetic anisotropy of the isatin moiety. According to the X-ray diffraction data, one of the *ortho*-protons is located almost above the centre of the isatin core. The estimated distance between the *ortho*-protons and the isatin fragment of approximately 4 Å corresponds to an NMR signal shift of 0.3–0.4 ppm.

According to this hypothesis, the dominant isomer **4i** is the structure in which the *ortho*-proton is located together with isatin on one side relative to the thiohydantoin plane, that is, isomer **B** in figure 3. In this case, although the *ortho*-fluorine atom in the major isomer of **4i** should also be more shielded, it should be taken into account that ^19^F nuclei have an incomparably wider range of chemical shifts than protons, and the difference in chemical shifts of 1.7 ppm observed for isomers **A** and **B** does not seem vital.

**Scheme 3.**
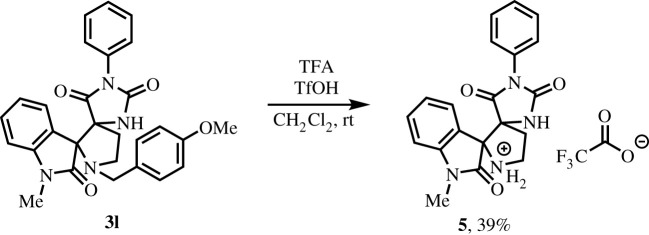
Removal of the *para*-methoxybenzyl group from the compound **3l**.

We also established that the *para*-methoxybenzyl group at the nitrogen atom of the central pyrrolidine ring of compound **3l** could be successfully removed by the action of a mixture of triflic and trifluoroacetic acids (scheme 3). Considering the higher solubility of the resulting compound **5** in water, this reaction is promising for further development of bioavailable spiro structures of this type. Notably, compound **5**, which has no hydrophobic substituents attached to the pyrrolidine nitrogen atom, unlike compounds **3** and **4**, was isolated from the aqueous phase in the form of a trifluoroacetate salt.

It should be noted that we were unable to conduct the reaction of 5-methylidene-2-chalcogenimidazolones with azomethine ylides generated from glycine and isatin. Even though there are examples in the literature of the addition of azomethineylides formed from alpha-amino acids and isatin in the presence of various additives [21,22]. In the absence of additives, the Schiff base formed in the course of the reaction is inefficiently attached to double bonds and is of no synthetic value. Thus, debenzylation of compound **3l** may be a convenient alternative method for the preparation of pyrrolidine-imidazolones, which do not contain substituents at the pyrrolidine nitrogen atoms.

## 3. Material and methods

### 3.1. General information

All common reagents were purchased from commercial suppliers and were used as received. The melting points were uncorrected. ^1^H (400 MHz), ^13^C (101 MHz) and ^19^F (376 MHz) NMR spectra were recorded on Bruker Avance 400 and Agilent MR-400 spectrometers in DMSO-d_6_ and C_6_D_6_. Chemical shifts were measured relative to residual solvent signals and referenced in parts per million to tetramethylsilane (TMS). High-resolution mass spectra (HRMS) were recorded on an OrbitrapElite (Thermo Scientific) mass spectrometer with electrospray ionization (ESI) and an orbital trap. To inject solutions with a concentration of 0.1 to 9 mg ml^−1^ (in 1% formic acid in acetonitrile), direct injection into the ion source using a syringe pump (0.3 ml min^−1^) was used. The spray voltage was ±3.5 kV, and the temperature of the capillary was 275°C.

Starting compounds **1** and **2a-i** were synthesized according to the methods described in [13].

CCDC 2052348 and 2052655 contain electronic supplementary material, crystallographic data for this study. These data can be obtained free of charge via www.ccdc.cam.ac.uk/data_request/cif, or by emailing data_request@ccdc.cam.ac.uk, or by contacting The Cambridge Crystallographic Data Centre, 12 Union Road, Cambridge CB2 1EZ, UK; fax: +44 1223 336033.

### 3.2. Synthetic procedures

#### 3.2.1. General procedure for the synthesis of dispiro compounds **3** and **4**

N-substituted amino acids (2 eq) were brought to reflux in methanol for hydantoin **1** or ethanol for thiohydantoins **2**. Subsequently, methylidene hydantoin **1** or methylidene thiohydantoin **2a**–**i** (1 eq) and the isatin derivative (2 eq) were added in one portion. The resulting mixture was refluxed for 4 h. After the reaction was completed, the reaction mixture was cooled to room temperature, the product was filtered off, washed with cold ethanol and dried in air. After filtration, the filtrate was evaporated under reduced pressure and the residue was purified by chromatography on silica gel to afford an additional amount of the desired dispiro compound (CHCl_3_ : MeOH, 40 : 1). The initial precipitate and the material after column chromatography were combined, and the overall yield was determined. All the products were crystalline solids.

#### 3.2.2. (2′R*,4S*)-1'-methyl-1-phenyldispiro[imidazolidine-4,3'-pyrrolidine-2′,3''-indoline]-2,2″,5-trione (**3a**)

From compound **1** (0.054 g, 0.26 mmol), isatin (0.078 g, 0.53 mmol) and sarcosine (0.047 g, 0.53 mmol) compound **3a** was obtained with the yield 0.046 g (51%) as a white crystalline solid. **^1^H NMR** (400 MHz, DMSO-d6): *δ* 10.54 (bs, 1H), 8.71 (bs, 1H), 7.43–7.39 (m, 2Н), 7.36 (d, *J* = 7.0 Hz, 1Н), 7.28 (t, *J* = 7.3 Hz, 1Н), 7.13 (d, *J* = 7.8 Hz, 1Н), 7.01-6.95 (m, 3Н), 6.84 (d, *J* = 7.4 Hz, 1Н), 3.31-3.26 (m, 2Н), 2.64-2.55 (m, 2Н), 2.04 (s, 3H). **^13^C NMR** (101 MHz, DMSO-d6): *δ* 176.2, 172.9, 154.8, 144.0, 131.7, 130.1, 128.8, 128.0, 126.9,123.8, 121.7, 109.9, 76.1, 70.6, 50.3, 35.4, 29.7. **HRMS** (ESI+) *m/z* calcd. for (C_20_H_18_N_4_O_3_, M + H): 363.1452, found: (M + H): 363.1454.

#### 3.2.3. (2′R*,4S*)-5″-fluoro-1'-methyl-1-phenyldispiro[imidazolidine-4,3'-pyrrolidine-2′,3″-indoline]-2,2″,5-trione (**3b**)

From compound **1** (0.054 g, 0.26 mmol), 5-fluoroisatin (0.087 g, 0.53 mmol) and sarcosine (0.047 g, 0.53 mmol) compound **3b** was obtained with the yield 0.055 g (55%) as a white crystalline solid. **^1^H NMR** (400 MHz, DMSO-d6): *δ* 10.60 (bs, 1H), 8.77 (bs, 1H), 7.43 (d, *J* = 7.5 Hz, 2Н), 7.37 (m, 1Н), 7.15 (m, 1Н), 6.97 (d, *J* = 8.0 Hz, 2H), 6.87-6.84 (m, 2Н), 3.29 (d, *J* = 6.9 Hz, 2Н), 2.63-2.53 (m, 2Н), 2.06 (s, 3H). **^13^C NMR** (101 MHz, DMSO-d6): *δ* 176.2, 172.7, 157.9 (d, *J*_CF_ = 237.5 Hz, 1C), 154.8, 140.2, 131.6, 128.9, 128.2, 126.7, 125.8 (d, *J*_CF_ = 7.4 Hz, 1C), 126.5 (d, *J*_CF_ = 22.9 Hz, 1C), 112.9 (d, *J*_CF_ = 25.8 Hz, 1C), 110.9 (d, *J*_CF_ = 7.6 Hz, 1C), 76.3, 70.9, 50.4, 35.4, 29.7. **HRMS** (ESI+) *m/z* calcd. for (C_20_H_17_FN_4_O_3_, M + H): 381.1357, found: (M + H): 381.1347.

#### 3.2.4. (2′R*,4S*)-5″-chloro-1'-methyl-1-phenyldispiro[imidazolidine-4,3'-pyrrolidine-2′,3″-indoline]-2,2″,5-trione (**3c**)

From compound **1** (0.054 g, 0.26 mmol), 5-chloroisatin (0.096 g, 0.53 mmol) and sarcosine (0.047 g, 0.53 mmol) compound **3c** was obtained with the yield 0.050 g (48%) as a white crystalline solid. **^1^H NMR** (400 MHz, DMSO-d6): *δ* 10.71 (bs, 1H), 8.79 (bs, 1H), 7.44 (m, 1Н), 7.42 (m, 1Н), 7.37 (d, *J* = 2.2 Hz, 1Н), 7.35 (d, *J* = 2.2 Hz, 1Н), 7.08 (d, *J* = 2.2 Hz, 1Н), 7.00 (m, 1Н), 6.98 (m, 1Н), 6.87 (d, *J* = 8.3 Hz, 1Н), 3.31-3.27 (m, 2Н), 2.63-2.54 (m, 2Н), 2.07 (s, 3H). **^13^C NMR** (101 MHz, DMSO-d6): *δ* 175.9, 172.8, 154.8, 142.9, 131.6, 130.0, 128.9, 128.3, 126.8, 126.0, 125.8, 125.3, 111.51, 76.2, 70.8, 50.4, 35.5, 29.5. **HRMS** (ESI+) *m/z* calcd. for (C_20_H_17_ClN_4_O_3_, M + H): 397.1062, found: (M + H): 397.1066.

#### 3.2.5. (2′R*,4S*)-7″-chloro-1'-methyl-1-phenyldispiro[imidazolidine-4,3'-pyrrolidine-2′,3″-indoline]-2,2″,5-trione (**3d**)

From compound **1** (0.054 g, 0.26 mmol), 7-chloroisatin (0.096 g, 0.53 mmol) and sarcosine (0.047 g, 0.53 mmol) compound **3d** was obtained with the yield 0.061 g (58%) as a white crystalline solid. **^1^H NMR** (400 MHz, DMSO-d6): *δ* 11.03 (bs, 1H), 8.80 (bs, 1H), 7.42-7.35 (m, 4Н), 7.10 (d, *J* = 6.7 Hz, 1Н), 7.04 (d, *J* = 7.9 Hz, 1Н), 7.00 (m, 1Н), 6.99 (m, 1Н), 3.31-3.28 (m, 2Н), 2.59 (m, 1Н), 2.48 (m, 1Н), 2.05 (s, 3H). **^13^C NMR** (101 MHz, DMSO-d6): *δ* 176.3, 172.6, 154.9, 141.6, 131.6, 130.3, 128.9, 128.1, 126.9, 125.9, 124.2, 123.1, 114.1, 76.8, 70.8, 50.3, 35.4, 29.7. **HRMS** (ESI+) *m/z* calcd. for (C_20_H_17_ClN_4_O_3_, M + H): 397.1062, found: (M + H): 397.1048.

#### 3.2.6. (2′R*,4S*)-1′,5″-dimethyl-1-phenyldispiro[imidazolidine-4,3'-pyrrolidine-2′,3″-indoline]-2,2″,5-trione (**3e**)

From compound **1** (0.054 g, 0.26 mmol), 5-methylisatin (0.086 g, 0.53 mmol) and sarcosine (0.047 g, 0.53 mmol) compound **3e** was obtained with the yield 0.045 g (45%) as a white crystalline solid. **^1^H NMR** (400 MHz, DMSO-d6): *δ* 10.44 (bs, 1H), 8.69 (bs, 1H), 7.43 (t, *J* = 7.4 Hz, 2Н), 7.37 (m, 1Н), 7.09 (d, *J* = 7.8 Hz, 1Н), 6.97-6.94 (m, 3Н), 6.73 (d, *J* = 7.8 Hz, 1Н), 3.31-3.26 (m, 2Н), 2.64-2.52 (m, 2Н), 2.20 (s, 3H), 2.04 (s, 3H). **^13^C NMR** (101 MHz, DMSO-d6): *δ* 176.1, 172.9, 154.8, 141.6, 131.8, 130.4, 130.3, 128.9, 128.1, 126.8, 126.0, 123.8, 109.7, 76.2, 70.6, 50.3, 35.5, 29.4, 20.7. **HRMS** (ESI+) *m/z* calcd. for (C_21_H_20_N_4_O_3_, M + H): 377.1608, found: (M + H): 377.1601.

#### 3.2.7. (2′R*,4S*)-1′,1″-dimethyl-1-phenyldispiro[imidazolidine-4,3'-pyrrolidine-2′,3″-indoline]-2,2″,5-trione (**3f**)

From compound **1** (0.054 g, 0.26 mmol), 1-methylisatin (0.086 g, 0.53 mmol) and sarcosine (0.047 g, 0.53 mmol) compound **3f** was obtained with the yield 0.062 g (62%) as a white crystalline solid. **^1^H NMR** (400 MHz, DMSO-d6): *δ* 8.68 (bs, 1H), 7.46-7.38 (m, 3H), 7.36 (m, 1H), 7.18 (d, *J* = 7.3 Hz, 1H), 7.11-7.03 (m, 2H), 6.97 (d, *J* = 7.5 Hz, 2H), 3.33-3.28 (m, 2H), 3.15 (s, 3H), 2.61 (m, 1H), 2.48 (m, 1H). **^1^H NMR** (600 MHz, C_6_D_6_): *δ* 7.66 (dd, ^3^*J* = 7.5 Hz, ^4^*J* = 1.2 Hz, 1H^14^), 7.27-7.26 (m, 2H^b^), 7.24-7.07 (m, 2H^c^), 7.01 (bs, 1H^2^), 6.90-6.95 (m, 1H^d^+1H^16^), 6.73 (dt, ^3^*J* = 7.5 Hz, ^4^*J* = 1.0 Hz, 1H^15^, 6.21 (bd, ^3^*J* = 7.7 Hz, 1H^17^), 3.505-3.45 (m, 1H^8^), 3.32-3.27 (m, 1H^8^), 2.67 (s, 3H^11^), 2.45-2.53 (m, 2H^9^), 2.01 (s, 3H^18^). **^13^C NMR** (151 МHz, C_6_D_6_): *δ* 176.1 (C^10^), 173.0 (C^5^), 155.3 (C^3^), 145.5 (C^12^), 132.5 (C^a^), 130.3 (C^16^), 128.9 (C^c^), 128.3 (C^d^), 126.9 (C^14^), 126.7 (C^b^), 124.1(C^13^), 123.3 (C^15^), 108.6 (C^17^), 77.5 (C^6^), 71.3 (C^1^), 51.2 (C^8^), 35.5 (C^18^), 31.9 (C^9^), 25.4 (C^11^). **^13^C NMR** (101 МHz, DMSO-d6): *δ* 176.4, 173.3, 155.6, 145.8, 132.8, 130.7, 129.3, 128.7, 127.2,127.1, 124.5, 123.6, 108.9, 77.8, 71.6, 51.5, 35.9, 32.2, 25.8. **HRMS** (ESI+) *m/z* calcd. for (C_21_H_20_N_4_O_3_, M + H): 377.1608, found: (M + H): 377.1620.

#### 3.2.8. (2′R*,4S*)-1'-methyl-1-phenyl-1″-(prop-2-yn-1-yl)dispiro[imidazolidine-4,3'-pyrrolidine-2′,3″-indoline]-2,2″,5-trione (**3g**)

From compound **1** (0.054 g, 0.26 mmol), 1-propargylisatin (0.098 g, 0.53 mmol) and sarcosine (0.047 g, 0.53 mmol) compound **3 g** was obtained with the yield 0.049 g (46%) as a white crystalline solid. **^1^H NMR** (400 MHz, DMSO-d6): *δ* 8.67 (bs, 1H), 7.41 (t, *J* = 7.5 Hz, 3Н), 7.36 (d, *J* = 7.2 Hz, 1Н), 7.22 (d, *J* = 7.7 Hz, 1Н), 7.13 (t, *J* = 8.5 Hz, 2Н), 6.98 (d, *J* = 7.8 Hz, 2Н), 4.56-4.54 (m, 2H), 3.30-3.27 (m, 2Н), 3.25 (m, 1H), 2.68-2.52 (m, 2Н), 2.00 (s, 3H). **^13^C NMR** (101 MHz, DMSO-d6): *δ* 173.9, 172.6, 154.8, 143.5, 131.6, 130.3, 128.8, 128.1, 126.8, 125.3, 123.1, 122.8, 109.7, 78.0, 75.9, 74.3, 70.6, 50.3, 35.16, 29.8, 28.5. **HRMS** (ESI+) *m/z* calcd. for (C_23_H_20_N_4_O_3_, M + H): 401.1608, found: (M + H): 401.1625.

#### 3.2.9. (2′R*,4S*)-1'-ethyl-1-phenyldispiro[imidazolidine-4,3'-pyrrolidine-2′,3″-indoline]-2,2″,5-trione (**3h**)

From compound **1** (0.054 g, 0.26 mmol), isatin (0.078 g, 0.53 mmol) and N-ethylglycine (0.055 g, 0.53 mmol) compound **3h** was obtained with the yield 0.038 g (39%) as a white crystalline solid. **^1^H NMR** (400 MHz, DMSO-d6): *δ* 10.49 (bs, 1H), 8.66 (bs, 1H), 7.43-7.31 (m, 3Н), 7.27 (t, *J* = 7.5 Hz,1Н), 7.16 (d, *J* = 7.2 Hz, 1Н), 7.02-6.94 (m, 3Н), 6.83 (d, *J* = 7.3 Hz, 1Н), 3.39 (m, 1Н), 3.22 (m, 1Н), 2.62 (m, 1Н), 2.44 (m, 1H), 2.35 (m, 1Н), 2.19 (m, 1Н), 0.90 (t, *J* = 6.3 Hz, 3H). **^13^C NMR** (101 MHz, DMSO-d6): *δ* 176.7, 172.7, 154.8, 143.9, 131.7, 130.0, 128.8, 128.0, 126.8, 125.5, 124.5, 121.6, 109.8, 76.0, 70.5, 47.8, 43.5, 29.3, 13.8. **HRMS** (ESI+) *m/z* calcd. for (C_21_H_20_N_4_O_3_, M + H): 377.1608, found: (M + H): 377.1599.

#### 3.2.10. (2′R*,4S*)-1'-(4-methoxybenzyl)-1-phenyldispiro[imidazolidine-4,3'-pyrrolidine-2′,3″-indoline]-2,2″,5-trione (**3i**)

From compound **1** (0.054 g, 0.26 mmol), isatin (0.078 g, 0.53 mmol) and *p*-methoxybenzyl glycine (0.104 g, 0.53 mmol) compound **3i** was obtained with the yield 0.035 g (28%) as a white crystalline solid. **^1^H NMR** (400 MHz, DMSO-d6): *δ* 10.56 (bs, 1H), 8.76 (bs, 1H), 7.46-7.39 (m, 2Н), 7.36 (m, 1H), 7.32-7.25 (m, 2Н), 7.22 (d, *J* = 8.1 Hz, 2Н), 7.06-7.00 (m, 2Н), 6.99 (m, 1H), 6.88-6.81 (m, 3Н), 3.71 (s, 3H), 3.33-3-24 (m, 2Н), 3.18-3.10 (m, 2Н), 2.69 (m, 1Н), 2.39 (m, 1Н). **^13^C NMR** (101 MHz, DMSO-d6): *δ* 176.5, 172.6, 158.3, 154.9, 144.2, 131.7, 130.4, 130.3, 128.8, 128.0, 126.8, 125.3, 124.1, 121.8, 113.7, 110.0, 75.6, 70.3, 55.0, 52.1, 47.8, 29.1. **HRMS** (ESI+) *m/z* calcd. for (C_27_H_24_N_4_O_4_, M + H): 469.1870, found: (M + H): 469.1871.

#### 3.2.11. (2′R*,4S*)-5″-chloro-1'-(4-methoxybenzyl)-1-phenyldispiro[imidazolidine-4,3'-pyrrolidine-2′,3″-indoline]-2,2″,5-trione (**3j**)

From compound **1** (0.054 g, 0.26 mmol), 5-chloroisatin (0.096 g, 0.53 mmol) and *p*-methoxybenzyl glycine (0.104 g, 0.53 mmol) compound **3j** was obtained with the yield 0.050 g (38%) as a white crystalline solid. **^1^H NMR** (400 MHz, DMSO-d6): *δ* 10.76 (s, 1H), 8.88 (s, 1H), 7.48-7.42 (m, 2H), 7.40 (d, *J* = 7.1 Hz, 1H), 7.35 (d, *J* = 8.7 Hz, 1H), 7.22-7.16 (m, 3Н), 7.03 (d, *J* = 7.76 Hz, 2Н), 6.89-6.82 (m, 3Н), 3.71 (s, 3H), 3.41 (d, *J* = 13.6 Hz,1Н), 3.33 (m, 1Н), 3.19-3.12 (m, 2Н), 2.66 (m, 1Н), 2.43 (m, 1Н). **^13^C NMR** (101 MHz, DMSO-d6): *δ* 176.2, 172.5, 158.3, 154.8, 143.0, 131.6, 130.2, 130.1, 128.9, 128.8, 128.2, 126.8, 126.3, 125.8, 125.0, 113.7, 111.6, 75.6, 70.6, 55.0, 52.3, 48.0, 28.9. **HRMS** (ESI+) *m/z* calcd. for (C_27_H_23_ClN_4_O_4_, M + H): 503.1481, found: (M + H): 503.1478.

#### 3.2.12. (2′R*,4S*)-5″-bromo-1'-(4-methoxybenzyl)-1-phenyldispiro[imidazolidine-4,3'-pyrrolidine-2′,3″-indoline]-2,2″,5-trione (**3k**)

From compound **1** (0.054 g, 0.26 mmol), 5-bromoisatin (0.120 g, 0.53 mmol) and *p*-methoxybenzyl glycine (0.104 g, 0.53 mmol) compound **3k** was obtained with the yield 0.046 g (32%) as a white crystalline solid. **^1^H NMR** (400 MHz, DMSO-d6): *δ* 10.77 (s, 1H), 8.88 (bs, 1H), 7.51-7.35 (m, 4Н), 7.40 (d, *J* = 7.0 Hz, 1Н), 7.30 (s, 1H), 7.19 (d, *J* = 7.5 Hz, 2Н), 7.05 (d, *J* = 7.4 Hz, 2Н), 6.90-6.78 (m, 3Н), 3.71 (s, 3H), 3.41 (d, *J* = 13.3 Hz, 1Н), 3.33 (m, 1Н), 3.20-3.15 (m, 2Н), 2.67 (m, 1Н), 2.42 (m, 1Н). **^13^C NMR** (101 MHz, DMSO-d6): *δ* 176.0, 172.6, 158.3, 154.8, 143.4, 132.9, 131.6, 130.2, 128.9, 128.8, 128.2, 127.8, 126.8, 126.6, 113.7, 113.5, 112.1, 75.6, 70.6, 55.0, 52.3, 48.0, 28.9. **HRMS** (ESI+) *m/z* calcd. for (C_27_H_23_BrN_4_O_4_, M + H): 547.0975, found: (M + H): 547.0978.

#### 3.2.13. (2′R*,4S*)-1'-(4-methoxybenzyl)-1″-methyl-1-phenyldispiro[imidazolidine-4,3'-pyrrolidine-2′,3″-indoline]-2,2″,5-trione (**3l**)

From compound **1** (0.054 g, 0.26 mmol), 1-methylisatin (0.086 g, 0.53 mmol) and *p*-methoxybenzyl glycine (0.104 g, 0.53 mmol) compound **3l** was obtained with the yield 0.057 g (45%) as a white crystalline solid. **^1^H NMR** (400 MHz, DMSO-d6): *δ* 8.75 (bs, 1H), 7.45-7.32 (m, 5Н), 7.19 (d, *J* = 8.53 Hz, 2Н), 7.11-7.01 (m, 4Н), 6.83 (d, *J* = 8.60 Hz, 2Н), 3.70 (s, 3H), 3.33 (m, 1Н), 3.28 (m, 1Н), 3.20-3.15 (m, 2Н), 3.17 (s, 3H), 2.69 (m, 1Н), 2.42 (m, 1Н). **^13^C NMR** (101 MHz, DMSO-d6): *δ* 174.7, 172.5, 158.3, 154.8, 145.5, 131.7, 130.4, 130.3, 128.8, 128.0, 126.8, 124.9, 123.4, 122.4, 113.6, 109.0, 75.3, 70.4, 55.0, 52.2, 47.9, 29.3, 26.0. **HRMS** (ESI+) *m/z* calcd. for (C_28_H_26_N_4_O_4_, M + H): 483.2027, found: (M + H): 483.2032.

#### 3.2.14. (2′R*,4S*)-1'-methyl-1-phenyl-2-thioxodispiro[imidazolidine-4,3'-pyrrolidine-2′,3″-indoline]-2″,5-dione (**4a**)

From compound **2a** (0.055 g, 0.27 mmol), isatin (0.079 g, 0.54 mmol) and sarcosine (0.048 g, 0.54 mmol) compound **4a** was obtained with the yield 0.054 g (53%) as a white crystalline solid. **^1^H NMR** (400 MHz, DMSO-d6): *δ* 10.61 (s, 1H), 10.60 (s, 1H), 7.43-7.37 (m, 3H), 7.32 (t, *J* = 7.6 Hz, 1H), 7.04 (d, *J* = 7.3 Hz, 1H), 6.97 (t, *J* = 7.5 Hz, 1H), 6.86 (d, *J* = 7.8 Hz, 1H), 6.84-6.79 (m, 2H), 3.44 (m, 1H), 3.31 (m, 1H), 2.67 (m, 1H), 2.52 (m, 1H), 2.03 (s, 3H). **^13^C NMR** (101 MHz, DMSO-d6): *δ* 182.4, 175.7, 173.5, 144.0, 133.1, 130.3, 128.8, 128.7, 128.3, 125.4, 123.4, 121.7, 110.0, 75.9, 73.2, 50.2, 35.1, 29.1. **HRMS** (ESI+) *m/z* calcd. for (C_20_H_18_N_4_O_2_S, M + H): 379.1223, found: (M + H): 379.1211.

#### 3.2.15. (2′R*,4S*)-5″-chloro-1'-methyl-1-phenyl-2-thioxodispiro[imidazolidine-4,3'-pyrrolidine-2′,3″-indoline]-2″,5-dione (**4b**)

From compound **2a** (0.055 g, 0.27 mmol), 5-chloroisatin (0.098 g, 0.54 mmol) and sarcosine (0.048 g, 0.54 mmol) compound **4b** was obtained with the yield 0.068 g (61%) as a white crystalline solid. **^1^H NMR** (400 MHz, DMSO-d6): *δ* 10.79 (s, 1H), 10.67 (bs, 1H), 7.47-7.37 (m, 4H), 6.98 (d, *J* = 2.0 Hz, 1H), 6.90 (d, *J* = 8.3 Hz, 1H), 6.86-6.82 (m, 2H), 3.34-3.29 (m, 2H), 2.68 (m, 1H), 2.54 (m, 1H), 2.06 (s, 3H). **^13^C NMR** (101 MHz, DMSO-d6): *δ* 182.4, 175.4, 173.4, 142.9, 133.0, 130.3, 128.9, 128.9, 128.6, 125.9, 125.6, 125.2, 111.6, 75.9, 73.4, 50.2, 35.2, 28.8. **HRMS** (ESI+) *m/z* calcd. for (C_20_H_17_ClN_4_O_2_S, M + H): 413.0834, found: (M + H): 413.0830.

#### 3.2.16. (2′R*,4S*)-1-(3-fluorophenyl)-1'-methyl-2-thioxodispiro[imidazolidine-4,3'-pyrrolidine-2′,3″-indoline]-2″,5-dione (**4c**)

From compound **2c** (0.055 g, 0.25 mmol), isatin (0.073 g, 0.50 mmol) and sarcosine (0.044 g, 0.50 mmol) compound **4c** was obtained with the yield 0.047 g (47%) as a white crystalline solid. **^1^H NMR** (400 MHz, DMSO-d6): *δ* 10.69 (s, 1H), 10.62 (s, 1H), 7.47 (m, 1H), 7.32 (td, *J* = 7.6, 1.3, 1H), 7.27 (td, *J* = 8.5, 2.3 Hz, 1H), 7.01 (d, *J* = 7.6 Hz, 1H), 6.96 (m, 1H), 6.86 (d, *J* = 7.7 Hz, 1H), 6.72 (dt, *J* = 9.5, 2.0 Hz, 1H), 6.65 (m, 1H), 3.33-3.30 (m, 2H), 2.66 (m, 1H), 2.54 (m, 1H), 2.04 (s, 3H). **^13^C NMR** (101 MHz, DMSO-d6): *δ* 181.9, 175.7, 173.2, 161.6 (d, *J*_CF_ = 244.4 Hz, 1C), 144.0, 134.6 (d, *J*_CF_ = 10.7 Hz, 1C), 130.4, 130.4 (d, *J*_CF_ = 8.8 Hz, 1C), 130.2, 125.3, 125.0 (d, *J*_CF_ = 3.0 Hz, 1C), 123.4, 121.7, 116.21, 115.9 (d, *J*_CF_ = 21.6 Hz, 1C), 110.1, 75.9, 73.4, 50.2, 35.1, 28.9. **HRMS** (ESI+) *m/z* calcd. for (C_20_H_17_FN_4_O_2_S, M + H): 397.1129, found: (M + H): 397.1126.

#### 3.2.17. (2′R*,4S*)-1-(4-fluorophenyl)-1′,1″-dimethyl-2-thioxodispiro[imidazolidine-4,3'-pyrrolidine-2′,3″-indoline]-2″,5-dione (**4d**)

From compound **2d** (0.055 g, 0.25 mmol), 1-methylisatin (0.074 g, 0.50 mmol) and sarcosine (0.044 g, 0.50 mmol) compound **4d** was obtained with the yield 0.064 g (64%) as a white crystalline solid. **^1^H NMR** (400 MHz, DMSO-d6): *δ* 10.63 (s, 1H), 7.43 (m, 1H), 7.26 (t, *J* = 8.9 Hz, 2H), 7.10-7.03 (m, 3H), 6.89-6.83 (m, 2H), 3.36-3.32 (m, 2H), 3.15 (s, 3H), 2.67 (m, 1H), 2.55 (m, 1H), 1.99 (m, 3H). **^13^C NMR** (101 MHz, DMSO-d6): *δ* 182.3, 173.9, 173.3, 161.8 (d, *J*_CF_ = 245.7 Hz, 1C), 145.3, 130.8 (d, *J*_CF_ = 9.0 Hz, 1C), 130.6, 129.3 (d, *J*_CF_ = 2.8 Hz, 1C), 124.8, 122.7, 122.4, 115.8 (d, *J*_CF_ = 22.9 Hz, 1C), 109.1, 75.6, 73.3, 50.2, 35.1, 29.1, 26.0. **HRMS** (ESI+) *m/z* calcd. for (C_21_H_19_FN_4_O_2_S, M + H): 411.1286, found: (M + H): 411.1279.

#### 3.2.18. (2′R*,4S*)-5″-bromo-1'-methyl-2-thioxo-1-(p-tolyl)dispiro[imidazolidine-4,3'-pyrrolidine-2′,3″-indoline]-2″,5-dione (**4e**)

From compound **2e** (0.055 g, 0.25 mmol), 5-bromoisatin (0.114 g, 0.50 mmol) and sarcosine (0.044 g, 0.50 mmol) compound **4e** was obtained with the yield 0.065 g (55%) as a white crystalline solid. **^1^H NMR** (400 MHz, DMSO-d6): *δ* 10.79 (s, 1H), 10.62 (s, 1H), 7.53 (d, *J* = 7.8 Hz, 1H), 7.23 (d, *J* = 7.8 Hz, 2H), 7.11 (s, 1H), 6.84 (d, *J* = 8.1 Hz, 1H), 6.73 (d, *J* = 7.7 Hz, 2H), 3.34-3.24 (m, 2H), 2.67 (m, 1H), 2.33 (s, 3H), 2.41-2.21 (m, 4H), 2.05 (s, 3H). **^13^C NMR** (101 MHz, DMSO-d6): *δ* 182.6, 175.2, 173.5, 143.3, 138.5, 133.1, 130.4, 129.4, 128.4, 127.9, 125.9, 113.3, 112.1, 75.8, 73.2, 50.2, 35.2, 28.7, 20.75. **HRMS** (ESI+) *m/z* calcd. for (C_21_H_19_BrN_4_O_2_S, M + H): 471.0485, found: (M + H): 471.0495.

#### 3.2.19. (2′R*,4S*)-1-(4-fluorophenyl)-1'-methyl-2-thioxodispiro[imidazolidine-4,3'-pyrrolidine-2′,3″-indoline]-2″,5-dione (**4f**)

From compound **2d** (0.056 g, 0.25 mmol), isatin (0.074 g, 0.50 mmol) and sarcosine (0.045 g, 0.50 mmol) compound **4f** was obtained with the yield 0.041 g (41%) as a light yellow crystalline solid. **^1^H NMR** (400 MHz, DMSO-d6): *δ* 10.62 (s, 1H), 10.60 (s, 1H), 7.32-7.23 (m, 3H), 7.02-7.00 (m, 1H), 6.97-6.93 (m, 1H), 6.88-6.83 (m, 3H), 3.36-3.25 (m, 2H), 2.68-2.61 (m, 1H), 2.54-2.49 (m, 1H), 2.02 (s, 3H). **^13^C NMR** (101 MHz, DMSO-d6): *δ* 182.7, 176.1, 173.8, 162.2 (d, *J*_CF_ = 245.7 Hz, 1C), 144.4, 131.2 (d, *J*_CF_ = 8.4 Hz, 1C), 130.8, 129.8 (d, *J*_CF_ = 3.1 Hz, 1C), 125.7, 123.8, 122.1, 116.1 (d, *J*_CF_ = 22.9 Hz, 1C), 110.5, 79.6, 76.3, 73.6, 50.8, 35.5. **HRMS** (ESI+) *m/z* calcd. for (C_20_H_17_FN_4_O_2_S, M + H): 397.1129, found: (M + H): 397.1134.

#### 3.2.20. (2′R*,4S*)-1-(4-methoxyphenyl)-1'-methyl-2-thioxodispiro[imidazolidine-4,3'-pyrrolidine-2′,3″-indoline]-2″,5-dione (**4g**)

From compound **2f** (0.056 g, 0.25 mmol), isatin (0.074 g, 0.50 mmol) and sarcosine (0.045 g, 0.50 mmol) compound **4g** was obtained with the yield 0.056 g (55%) as a light yellow crystalline solid. **^1^H NMR** (400 MHz, DMSO-d6): *δ* 10.58 (s, 1H), 10.51 (s, 1H), 7.30 (dt, *J*_1_ = 1.0 Hz, *J*_2_ = 7.6 Hz, 1H), 7.03-7.01 (m, 1H), 6.97-6.92 (m, 3H), 6.84 (m, *J* = 7.7 Hz, 1H), 6.70 (d, *J* = 8.7 Hz, 2H), 3.75 (s, 3H), 3.34-3.26 (m, 2H), 2.68-2.61 (m, 1H), 2.49-2.44 (m, 1H), 2.02 (s, 3H). **^13^C NMR** (101 MHz, DMSO-d6): *δ* 182.8, 175.8, 173.7, 159.2, 144.0, 130.3, 129.8, 125.7, 125.4, 123.4, 121.6, 114.0, 100.0, 75.9, 73.1, 55.4, 50.2, 35.1, 29.1. **HRMS** (ESI+) *m/z* calcd. for (C_21_H_20_N_4_O_3_S, M + H): 409.1329, found: (M + H): 409.1347.

#### 3.2.21. (2′R*,4S*)-1'-methyl-2-thioxo-1-(m-tolyl)dispiro[imidazolidine-4,3'-pyrrolidine-2′,3″-indoline]-2″,5-dione (**4h**)

From compound **2g** (0.056 g, 0.25 mmol), isatin (0.074 g, 0.50 mmol) and sarcosine (0.045 g, 0.50 mmol) compound **4h** was obtained with the yield 0.053 g (54%) as a light yellow crystalline solid. **^1^H NMR** (400 MHz, DMSO-d6): *δ* 10.60 (s, 1H), 10.56 (s, 1H), 7.34-7.25 (m, 2H), 7.19-7.17 (m, 1H), 7.03-7.01 (m, 1H), 6.98-6.94 (m, 1H), 6.85 (d, *J* = 7.7 Hz, 1H), 6.60-6.55 (m, 2H), 3.35-3.26 (m, 2H), 2.67-2.61 (m, 1H), 2.52-2.46 (m, 1H), 2.26 (s, 3H), 2.03 (s, 3H). **^13^C NMR** (101 MHz, DMSO-d6): *δ* 182.4, 175.8, 173.6, 144.0, 138.2, 133.1, 130.2, 129.4, 129.1, 128.6, 125.8, 125.5, 123.4, 121.6, 110.0, 75.9, 73.2, 50.2, 35.1, 30.7, 20.8. **HRMS** (ESI+) *m/z* calcd. for (C_21_H_20_N_4_O_2_S, M + H): 393.1380, found: (M + H): 393.1383.

#### 3.2.22. (2′R*,4S*)-1-(2-fluorophenyl)-1'-methyl-2-thioxodispiro[imidazolidine-4,3'-pyrrolidine-2′,3″-indoline]-2″,5-dione (**4i**)

From compound **2h** (0.056 g, 0.25 mmol), isatin (0.074 g, 0.50 mmol) and sarcosine (0.045 g, 0.50 mmol) compound **4i** was obtained with the yield 0.045 g (45%) as a light yellow crystalline solid. **^1^H NMR** (400 MHz, DMSO-d6; see also electronic supplementary material, Information, pp. S44–S46): *δ* 10.85 (s, 1H^2^), 10.68 (s, 1H^11^), 10.65 (s, 1H^2′^), 10.60 (s, 1H^11′^), 7.60-7.34 (m, 1H^d^+1H^d′^+1H^16^+1H^f′^), 7.30-7.24 (m, 1H^16′^+1H^e^+1H^e′^+1H^c′^), 7.02-6.98 (m, 1H^15^+1H^14^+1H^14′^), 6.88-6.85 (m, 1H^17^+1H^15′^), 6.83 (d, *J* = 7.7 Hz, 1H^17′^), 6.40 (td, *J*_1_ = 1.6 Hz, *J*_2_ = 7.7 Hz, 1H^f^), 3.35-3.26 (m, 2H^8^+2H^8′^), 2.73-2.65 (m, 1H^9^+1H^9′^), 2.58-2.53 (m, 1H^9′^), 2.43-2.35 (m, 1H^9^), 2.06 (s, 3H, Me), 2.03(s, 3H, Me^′^). **^13^C NMR** (101 MHz, DMSO-d6): *δ* 182.2 (С^3′^), 181.9 (C^3^), 176.0 (C^10^), 176.0 (C^10′^), 173.5 (C^5′^), 173.5(C^5^), 159.2 (d, *J*_CF_ = 250.0 Hz, C^b^), 158.8 (d, *J*_CF_ = 253.0 Hz, C^b′^), 144.4 (C^12^), 144.0 (C^12′^), 131.7 (d, *J*_CF_ = 8.0 Hz, C^16′^), 131.2 (d, *J*_CF_ = 7.9 Hz, C^d′^), 130.4 (d, *J*_CF_ = 12.5 Hz, C^f^ +C^f′^), 130.0 (C^16^), 125.7 (C^14′^), 125.2 (C^14^), 124.9 (d, *J*_CF_ = 3.6 Hz, C^e^), 124.5 (d, *J*_CF_ = 3.6 Hz, C^e′^), 123.2 (C^13^ + C^13′^), 121.9 (C^15′^), 121.8 (C^15^), 120.7 (d, *J*_CF_ = 13.4 Hz, C^a′^), 120.5 (d, *J*_CF_ = 12.8 Hz, C^a^), 116.4 (d, *J*_CF_ = 19.3 Hz, C^c^), 116.1 (d, *J*_CF_ = 19.1 Hz, C^c′^), 110.2 (C^17^), 109.7(C^17′^), 75.9 (C^6^), 75.5(C^6′^), 73.6 (C^1^), 73.5 (C^1′^), 50.1 (C^8^), 49.9 (C^8′^), 35.1 (Me), 35.1 (Me^′^), 30.1 (C^9′^), 29.1 (C^9^). **^19^F NMR** (376 MHz, DMSO-d6): −118.70 and −120.59. **HRMS** (ESI+) *m/z* calcd. for (C_20_H_17_FN_4_O_2_S, M + H): 397.1129, found: (M + H): 397.1135.

#### 3.2.23. (2^′^R*,4S*)-1-(2-chlorophenyl)-1'-methyl-2-thioxodispiro[imidazolidine-4,3'-pyrrolidine-2′,3″-indoline]-2″,5-dione (**4j**).

From compound **2i** (0.060 g, 0.25 mmol), isatin (0.074 g, 0.50 mmol) and sarcosine (0.045 g, 0.50 mmol) compound **4j** was obtained with the yield 0.048 g (47%) as a light yellow crystalline solid. **^1^H NMR** (400 MHz, DMSO-d6): *δ* 10.76 (s, 1H), 10.62 (s, 1H), 10.52 (s, 1H^′^), 10.49 (s, 1H^′^), 7.60-6.75 (m, 3H+4H^′^), 6.36 (dd, ^3^*J* = 7.7 Hz, ^4^*J* = 1.5,Hz, 1H), 3.35-3.19 (m, 2H+2H^′^), 2.73-2.59 (m, 1H+2H^′^), 2.38-2.31 (m, 1H^′^), 2.01 (s, 3H), 1.98 (s, 3H^′^). **^13^C NMR** (101 MHz, DMSO-d6): *δ* 182.0, 181.8, 176.2, 176.0, 173.4, 173.2, 144.4, 144.2, 138.8, 133.3, 133.0, 132.2, 131.7, 131.3, 131.1, 131.0, 130.9, 130.4, 130.1, 128.5, 128.1, 127.1, 125.7, 125.1, 124.0, 123.6, 123.2, 122.5, 122.2, 112.6, 110.6, 110.1, 79.6, 79.1, 74.1, 74.0, 50.5, 35.5, 35.4, 31.6, 29.6. **HRMS** (ESI+) *m/z* calcd. for (C_21_H_19_ClN_4_O_2_S, M + H): 413.0839, found: (M + H): 413.0840.

#### 3.2.24. General procedure for the synthesis of (2′R*,4S*)-1″-methyl-1-phenyldispiro[imidazolidine-4,3'-pyrrolidine-2′,3″-indoline]-2,2″,5-trionetrifluoroacetic acid salt **5**

This compound was obtained according to a procedure similar to that described in [23]. To a solution of compound **7l** (0.092 g, 0.19 mmol) in dichloromethane (5 ml), 1.4 ml of trifluoroacetic acid and 77 µl of triflic acid were added and the mixture was stirred at room temperature. After 8 h, another 77 µl of triflic acid was added and the mixture was stirred for 48 h. After that, the reaction mixture was poured into water and extracted with 20 ml × 2 dichloromethane, the organic fractions were discarded, and the aqueous phase was evaporated to dryness. The yield of 90.035 g (39%) as a white spongy substance. **^1^H NMR** (400 MHz, D_2_O): *δ* 7.66 (td, *J* = 7.9, 1.2 Hz, 1H), 7.56-7.53 (m, 3H), 7.43 (m, 1H), 7.28 (td, *J* = 7.7, 0.9 Hz, 1H), 7.25 (d, *J* = 7.9 H, 1H), 7.07-7.04 (m, 2H), 4.15 (m, 1H), 4.09 (m, 1H), 3.33 (s, 3H), 3.14 (m, 1H), 2.90 (m, 1H). **^13^C NMR** (101 MHz, D_2_O): *δ* 173.5, 170.8, 156.8, 144.8, 133.3, 130.1, 129.9, 129.3, 126.9, 125.2, 124.3, 119.7 (q, *J* = 317.3 Hz, 1C), 116.9, 111.0, 71.1, 69.0, 43.7, 28.1, 26.7. **HRMS** (ESI+) *m/z* calcd. for (C_20_H_19_N_4_O_3_): 363.1452, found: (M + H): 363.1463.

## 4. Conclusion

This paper describes a new and convenient synthesis of dispiro indolinone-pyrrolidine-imidazolones from 5-methylidene derivatives of hydantoins and thiohydantoins, N-substituted glycines and isatines by means of diastereoselective 1,3-dipolar cycloaddition. For dipolarophiles with *ortho*-substituted benzene fragments at the N(3) nitrogen atom of the 2-thiohydantoin rings 4i and 4j, the reaction takes place with the formation of two atropisomers due to the rotation barrier around the Nsp^2^–Caryl bond. For the dispiro derivative 3l, containing a *para*-methoxybenzyl substituent on the nitrogen atom of the central pyrrolidine ring, the possibility of transformation into the corresponding water-soluble N-pyrrolidine-unsubstituted analogue was shown.

## Data Availability

All data collected as part of this study are available from the Dryad Digital Repository: https://doi.org/10.5061/dryad.6t1g1jx0v [24]. The data are provided in the electronic supplementary material [25].
